# In Vitro Techniques and Measurements of Phage Characteristics That Are Important for Phage Therapy Success

**DOI:** 10.3390/v14071490

**Published:** 2022-07-07

**Authors:** Tea Glonti, Jean-Paul Pirnay

**Affiliations:** Laboratory for Molecular and Cellular Technology, Queen Astrid Military Hospital, B-1120 Brussels, Belgium; jean-paul.pirnay@mil.be

**Keywords:** phage isolation, phage selection, phage virulence, phage activity, phage therapy

## Abstract

Validated methods for phage selection, host range expansion, and lytic activity determination are indispensable for maximizing phage therapy outcomes. In this review, we describe some relevant methods, highlighting their advantages and disadvantages, and categorize them as preliminary or confirmatory methods where appropriate. Experimental conditions, such as the composition and consistency of culture media, have an impact on bacterial growth and, consequently, phage propagation and the selection of phage-resistant mutants. The phages require different experimental conditions to be tested to fully reveal their characteristics and phage therapy potential in view of their future use in therapy. Phage lytic activity or virulence should be considered as a result of the phage, its host, and intracellular/environmental factors, including the ability of a phage to recognize receptors on the bacterial cell surface. In vitro quantitative and qualitative measurements of phage characteristics, further validated by in vivo experiments, could be incorporated into one system or mathematical model/formula, which could predict a potential successful outcome of clinical applications.

## 1. Introduction

### 1.1. What Is a Phage?

A century ago, bacteriophages (phages) were defined as “devourers of bacteria” [1] or “obligate intracellular parasites” [2]. Soon after their discovery, and still today in Post-Soviet states [3] and their European satellites, they were used as antibacterial agents in medicine, but in the rest of Europe and the United States (US) they were relegated to the background upon the marketing of antibiotics in the 1940s. However, even today, we still have not fully grasped the complex biology of phages and their interactions with both bacterial hosts and mammalian immune system [4]. Upon their rediscovery by Western medicine, phages were classified as medicinal products (European Union) or drugs (US), without providing a dedicated framework for their development, marketing, and clinical application. As such, regulators underappreciated a number of peculiarities phages have with respect to conventional antibacterials, such as their narrow host specificity and antagonistic coevolution with these hosts [5]. In addition, phages are increasingly being played off as nano-carriers, delivering an engineered or armed DNA/RNA bactericidal payload [6], while replicating and evolving in and with bacteria.

Phages are the most abundant and diverse life-like entities on Earth, where they are found in almost all ecospheres, such as seas, rivers, and soil, and within other organisms, including humans. They control the abundance of their bacterial hosts and, as such, also impact global energy and nutrient cycles [4]. Phages can also affect host diversity, e.g., by “killing the winner”, and this keeps competitively dominant species or populations “in check” [7]. As such, they may be employed for the biological control of environments [8,9]. It is exactly this characteristic of phages that should be considered when “domesticating” them to control infecting or contaminating bacteria in patients, agriculture, or food processing. Today, a number of phage products are used in the agro-food industry, for instance, as bio-sanitation agents on ready-to-eat foods [10].

### 1.2. What Is Phage Virulence?

The words “virulence” and “virulent” come from the Latin word *virulentus*, meaning “full of poison”. They are used to indicate the relative capacity of a “microbe” (bacterium, fungus, or virus) to cause disease [11], or in classical microbiological manuals, to describe a degree of pathogenicity. Translated to phages, virulence could thus be defined as a degree of lytic (causing or resulting from lysis) activity at a given condition. In the specialized scientific literature, however, phage virulence is often employed to indicate phages that undertake lytic rather than lysogenic cycles [12,13]. Lytic (virulent) phages own the ability of self-replication and high specificity against target bacteria [14]. Gill et al. apply the term “virulence” to indicate the potential of a phage strain to drive specific bacterial cultures to extinction (or, at least, to very low densities) [15]. Phage virulence can also be defined as the ability of a phage to control the growth of its host in culture (culture clearing) [16], and may also be an indicator of phage utility [17]. Sometimes it is linked to the phage’s burst size as a prerequisite for productive-infection treatment [18].

In fact, virulence is not a distinct phage characteristic, but a complex, dynamic, and variable phenomenon that includes both phage and bacterial factors [11]. Indeed, it would be difficult to consider phage virulence as a single parameter, as phage-host interactions could range from the partial to total elimination of the targeted bacterial population. At the same time, complete lysis depends on the host/population and specific conditional factors as well. Phage virulence should be defined as a set of phage characteristics and ambient factors that effect, in a supportive manner, phage lytic activity levels or, in other words, the relative capacity to produce dynamic and high levels of bacterial lysis. Phage virulence levels could be extended by efficiently controlling phage/bacteria interactions, e.g., under rationally developed in vitro conditions.

### 1.3. The Challenge

Nowadays, experts increasingly agree that phages will not replace antibiotics [19], and could sometimes be more effective when used in combination with (sub-inhibitory concentrations of) antibiotics [20]. For instance, combinations of phages and antibiotics were shown to be more potent in killing *Pseudomonas aeruginosa* than either one acting alone [21]. Phages could thus be considered as supportive therapeutics to facilitate the management of relevant infectious diseases or complications. The lack of basic understanding of phage biology is considered to be [22] one of the causes for phage therapy failures in the early days. Because bacteria represent an environmental community for, and a hosting facility to, phages, fundamental studies analyzing the interactions between phages and bacteria [23], and predicting the dynamics between phage and bacterial populations [24], are of paramount importance [25,26,27] to developing practical phage therapy approaches.

Today’s laboratory facilities and materials are more developed than those in Félix d’Hérelle’s time. Glass tubes and Pasteur pipettes, for instance, are replaced with Eppendorf tubes or 96-well microtiter plates and multichannel micropipettes. Notwithstanding the modernization of laboratory equipment, there are no significant differences in the techniques used for phage isolation and propagation, the development of phage cocktails, nor the (large-scale) production of therapeutic phage preparations. In 1930, d’Hérelle recognized that the most effective therapeutic phages could be isolated from patients that had recovered from infection. He also claimed that more than 50 bacterial strains should be used in phage isolation and enrichment methods [28]. Interestingly, adapted versions of two of d’Hérelle’s phage cocktail formulations (Pyophage and Intestiphage) are still predominantly used in Georgia and Russia today [20]. It is very important to balance the growth rate of phages and bacteria, creating the optimal conditions for their productive interactions. In 1966, Thomas and Abelson observed that for optimal phage propagation, bacterial cultures should be “growing logarithmically at the time of infection”. In 1970, Sargeant demonstrated the importance of a good supply of living bacteria and aeration for obtaining a large quantity of phages [29]. In 1980, David et al. used a *Mycobacterium smegmatis* “surrogate” strain for the propagation of *M. tuberculosis* phages [28] to improve the practicality of procedures and to comply with biosafety requirements. In 1992, Yin and McCaskill observed the importance of maintaining the balance between the growth rate of bacteria and the phage. In one particular case, they showed that “slowing down” phage plaque formation (phage particle diffusing rate) to pace bacterial growth resulted in higher phage concentrations expressed in plaque forming units (pfu)/mL [4]. Notwithstanding these observations, we are still a long way from a full understanding of the etiology of phage/bacteria interactions [30]. Several recent review papers have considered the existing skills and expertise with regard to phage research and their medical use. There is a consensus that screening and selecting the right phages is of key importance for achieving successful therapeutic outcomes. Some suggest that the impact of phages on bacterial biofilms could be crucial toward understanding both phage and bacterial ecology [9]. However, the challenge is that there are no validated in vitro methods [31,32] to determine the phage characteristics that are important for predicting in vivo therapeutic efficacy [22] or performance [27], for instance, in view of future clinical trials that are desperately needed both to prove phage product efficacy and to determine the most effective phage therapy protocols [20].

This review brings together relevant methods for phage isolation, detection, characterization, and selection, including phage activity determination, host range evaluation and expansion, and the translation of in vitro results to clinical practice. We will mostly focus on the practical side of these methods (technical protocols), including some inputs and interpretations based on our personal experiences, as well as the advantages and disadvantages of the methods with regard to developing more standardized approaches.

## 2. In Vitro and In Vivo Phage Detection and Phage Activity Testing

In this section, we will discuss a number of methods that are commonly used for in vitro phage lytic activity determination, including phage detection and enumeration testing and the in vivo translation of results (Diagram 1).

### 2.1. Phage Isolation Enrichment Method and Bacteria Hooks

Bacterialstrains used for the “fishing” or detection of new phages are referred to here as “bacteria hooks”. For the isolation of potentially new phages, the well-known “phage enrichment” (PE) method is used. It was first developed by Winogradsky and Beijerinck [33] and later adapted by Jassim et al. [34] and Jensen et al. [16,28]. An updated version of the protocol was described by Twest and Kropinski [35] and by Merabishvili et al. [36], both in 2009. PE sometimes implies involving a larger bacterial panel BP [8] of potential “bacteria hooks”, as this facilitates the rapid isolation of polyvalent phages from the environment [37]. The use of an enrichment BP increases the possibility of catching a larger variety of phages in a given sampling source and can also increase phage titers, which facilitates the detection of potentially new phages. The best practice is to develop an enrichment BP for each bacterial species separately (homogeneous matrix), but a heterogeneous approach can also be used. A homogeneous enrichment BP should ideally consist of:

- Bacteria hooks with hosts covering the wide range of receptors needed to hook the largest variety of potential phages. This requires having a readily available panel of strains with known genetic profiles. Every newly isolated phage can be further studied, e.g., to determine its biology;
- Bacteria hooks of particular interest can be included. In this case, bacterial strains are selected based on specific features such as antibiotic resistance, and it is not necessary to have an exhaustive list of characteristics or to know their genomic profile. The strains could be objects of further scientific study.

**Figure A1:**
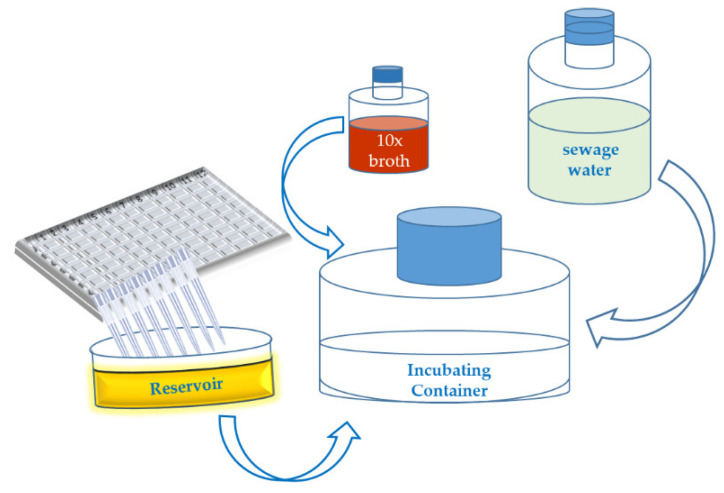
Diagram depicting the “phage isolation enrichment” method.

Bacteria hooks consisting of working host strains, i.e., strains that have already been adapted/approved for phage propagation/production, speed up downstream phage adaptation/training procedures. Newly isolated phages could, of course, also be propagated and trained in other bacterial strains than the ones used for isolation. A scaled-up version of the PE approach is described by Olsen et al. as part of a high-throughput screening (HiTS) method for phages. They propose using 96-deep-well plates, which allows for the simultaneous handling of a large range of environmental samples (water). One single host is used in each well containing 1.5 mL of water sample, and the method is oriented towards predominantly lytic and easily cultivable phages [38]. An outline of a PE method that uses a large number (96 or 384) of bacteria hooks is described in Appendix A, Figure A1. The technique is less time-, material-, and labor-consuming. It uses a large number of bacteria hooks in a relatively small volume and multichannel pipetting. This approach makes it easier to contain the infectious material advised for Biosafety and Biosecurity reasons. Water (sewage, river, lake) or liquefied soil and clinical samples/materials can be used as potential sampling sources for phages. In short:

- Two times [35] or ten times [36] concentrated broth medium is typically added to the phage-sampling source to ensure sufficient nutrition. When using large sampling volumes, it is rational to use more concentrated (up to 20 times) broth media that will generate less volume of the end product, which makes it easier and safer when handling infectious material;
- It is preferable not to centrifuge/filter the sampling source, unless it contains large contaminants and/or components that will interfere with the incubation process. It is assumed that conditions close to those in the natural source environment will facilitate phage/bacteria interactions and the isolation of phages;
- Using lower temperatures (25–28 °C) than those routinely used in clinical microbiology (30–37 °C) [35,36] and longer incubation times, for instance 24 h (where commonly 4–6 h is enough for phage propagation in liquid media), are more favorable for PE. However, long incubation periods could also have an adverse effect on phage particles. Because the ratio of phage emergence to bacteria (those initially present in the sample and the added bacteria hooks) in the enrichment propagation mixture is not preliminary determined as obtaining consistent lysis without early (e.g., <24 h) phage-resistant bacterial mutant growth or phage antagonistic activity. In addition, some bacterial products could interfere with phage propagation or the demonstration of phage activity;
- Using 96- or 384-well microtiter plates for the incubation of a large number of inoculums of bacteria hooks is more convenient. The bacterial suspensions are collected from each well using a multichannel pipette (Appendix A, Figure A1);
- After incubation, the potential phage lysate (PL) is centrifuged and filtered. There is no necessity for the use of chloroform, as this could reduce the viruses’ infectivity [39] or inactivate some phages [16] and could also lead to the induction of temperate phages [40]. Using chloroform is a tradition that dates back to the time when bacterial filters were not available, and the procedure itself was not enough to ensure absolute removal of bacterial contamination. Adding the right amount (0.5–2% *v*/*v*) of chloroform to PL at +4 °C (temperature shock) kills the remaining intact bacterial cells, including lyrically phage-infected bacteria, and could thus result in substantially increased phage titers [16]. Chloroform was also used for the medium term (3–12 months) storage of phage stocks, as it prevented bacterial growth [41]. In addition to the obvious laboratory personnel safety issues (hazardous chemicals), it is not recommended to use chloroform for phage preparations that will be used in clinical treatments;
- The obtained PL could be used further as the second source for another enrichment BP with different bacteria hooks.

It is considered a disadvantage of the PE method that faster-growing phages will outcompete phages with slower-growing populations [42], masking the appearance of potentially interesting phages (e.g., broader host-ranges) [15,42]. 

#### Phage Detection—Preliminary Tests

Generally, the PE lysate is first tested against the bacteria hooks used in the PE method, but it could also be carried out using any other relevant BP, for instance, containing strains from available bacterial culture collections [18]. Different methods are used for the detection of new phages in the lysates.

(i) The “direct spot test” (here, we call it a technique): in which only one dilution of the phage lysate is spotted on bacteria grown directly on solid agar. It is described below;
(ii) The “spot test” [43] (we will further use this name for a technique): in which one dilution of the phage lysate is spotted on a film of bacteria growing in a “top agar” surface [44]. This technique is also called “spot testing” [21] or “direct spot” [45];
(iii) The “lysis profile assay” [21] or, as we call it here, “phage liquid culturing” (PLC) method implies the liquid culture of phage/bacteria mixtures at specific dilution(s) in microtiter plates for the determination of phage susceptibility. As many as 5- to 10-fold greater numbers of bacterial test strains could be considered per microtiter plate, as compared to the conventional “spot tests” performed on petri dishes of different sizes and shapes [28]. This results in reduced hands-on time and fewer consumables.

**Figure A2:**
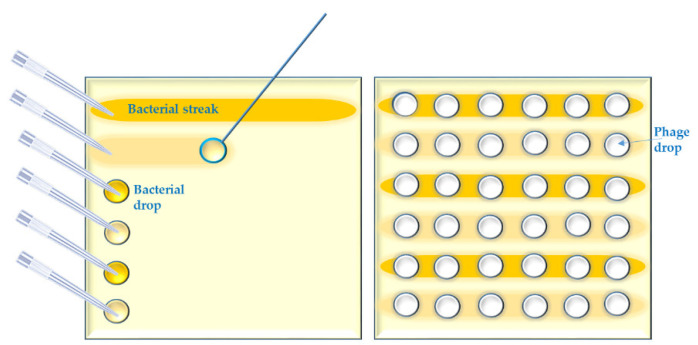
Diagram depicting the “spot on streak” method.

**Figure A3:**
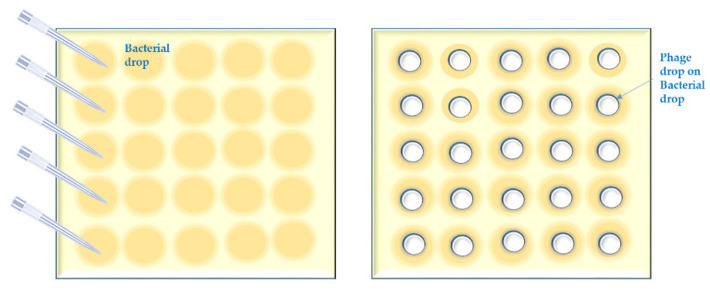
Diagram depicting the “spot on spot” method.

**Figure A4:**
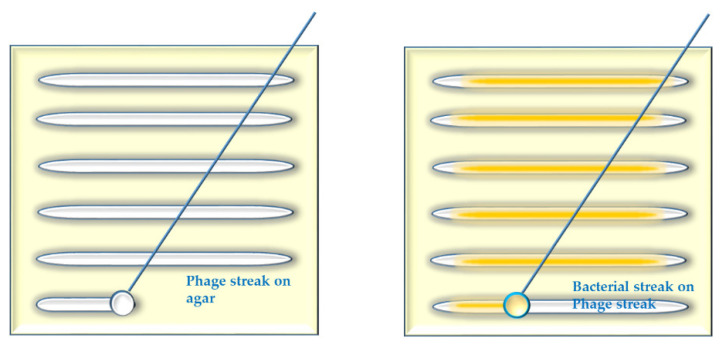
Diagram depicting the “streak on streak” method.

In the “direct spot test”, bacteria can be grown either as a series of distinct areas (streaks or spots) or as complete lawns on solid agar (without soft agar overlay). Phage lysates are applied in the areas of expected bacterial growth. Bacteria are commonly applied in three ways:

- Several parallel streaks (“streak assay” [36,46]) of bacterial suspension(s) of particular dilution(s) are made using disposable loops (Appendix A, Figure A2). Phage lysate(s) are applied as spots on the bacterial streaks (we call it “spot-on-streak” to differentiate from the other techniques);
- Bacterial suspensions are simply spotted [47] in a grid. Phage lysate(s) are applied as spots (we call it “spot-on-spot”) (Appendix A, Figure A3);
- Bacterial suspensions are directly streaked on streaks of phages made on solid agar [48] (we call it “streak-on-streak”) (Appendix A, Figure A4).

The first two preliminary phage detection approaches allow for the screening of large numbers of BPs and phages. The choice between either of them is a matter of practicality.

It is considered that the “spot-on-streak” assay (a variation of the “direct spot test”) does not allow for the evaluation of a possible emergence of bacterial phage resistance [34]. In fact, the “streak assay” does not allow for the study of phage kinetics. However, it does allow for a qualitative assessment of the tendency towards bacterial phage resistance through the visual observation of phage resistant mutants that emerge as individual colonies or confluent growth over the clear (lysis) zones of spotting. Bacterial colonies isolated from bacterial “over-growth” on agar plates or liquid samples taken from PLC “re-growth” need to be further tested to confirm that over- or re-growth [34] is indeed due to phage resistant bacterial mutants.

All the previously mentioned methods should be considered as preliminary detection techniques, as they are merely revealing bacterial lysis on agar or in liquid and do not confirm that these are the result of phage activity.

As PE lysates potentially consist of different phage variants at different concentrations, possibly including rare and interesting variants at low titers, it is reasonable to continue evaluating the PE lysates without diluting them. Bacterial suspensions used in the above-mentioned methods should have a minimum concentration of 10^4^ colony-forming units (cfu)/mL, which will result in sufficient growth to reveal the activity of phages that are present at a low concentration. The “spot-on-streak” assay allows for the application of multiple phage lysates on multiple bacterial strains, at different dilutions, on one plate. Note that bacteria grow slower on a solid agar surface than in broth, which will help phages that are present at lower titers, or with slower reproduction rates, to pace the bacterial growth and reveal themselves. Moreover, when large-size phage virions cannot diffuse [28] in soft agar, they find it easier to proliferate on low-density bacterial growth directly on the solid agar surface. In addition, it is easier to handle than modifying the soft agar method by using 0.2% (wt/vol) low melting point agarose [49], thus increasing the possibility of the diffusion of phage particles and, correspondingly, improving plaque formation. Another approach to detect low numbers of phages is using sub-lethal doses of antibiotics (e.g., 2.5–3.5 mg/mL of ampicillin, depending on the agar concentration of the top layer), which helps the formation of visible plaques [28].

Pipetting robots could be used for the “spot-testing”-based methods. A rectangular-shaped tray-plate, from SPL life science, for instance, is perfect to perform the spotting and could be fixed on the pipetting robot workstation. The advantage of that plate is that it has nearly the same dimensions (127.94 × 85.50 mm) as a 96-well microtiter plate (127.71 × 85.43 mm), which can be used as a reservoir for the phages that will be spotted. The spotting height should be adjusted correctly to avoid piercing the agar surface or splashing the drop while spotting, and thus generating aerosols and subsequent cross-contamination. In case of the “spot-on-streak” assay, bacterial streaks are pre-prepared, while the “spot-on-spot” method could be performed entirely by the pipetting robot.

After visual examination of the lysis zones and interpretation of the preliminary results, several phage/host bacteria combinations are selected to be further submitted to confirmatory methods that are able to reveal true phage plaque formation.

### 2.2. Confirmatory Test for Phage Activity Detection/Enumeration—Plaque Formation

Plaque formation is the result of multiple rounds of infection, lysis, and release of progeny [18], and it varies according to the phage’s latent period, burst size, diffusion rate and host bacterial growth; all these parameters are finally revealed in different plaque sizes and visibilities [17,50,51].

While a variety, or the technical modification, of methods are used for plaque formation and enumeration, double agar layer (DAL) methods are the most commonly used.

The main reasons for using plaque formation assays are:

- Confirmation of plaque formation;
- Study of plaque morphology;
- Enumeration (determination of pfu/mL) of phages.

The morphological appearance of the individual plaques is the first parameter that needs to be determined, as it is of great importance for:

- Phage differentiation/selection;
- Plaque purification;
- Phage virulence/lysogeny evaluation procedures.

#### 2.2.1. Double Agar Layer (DAL) Method

The DAL method was independently developed by the Belgian microbiologist André Gratia in 1936 (“Des relations numériques entre bactéries lysogènes et particules de bactériophage”), and by Hershey, Kalmanson, and Bronfenbrenner in 1943 [46], to be formalized later by Adams in 1959 [33]. An updated version (Double Agar Overlay Plaque Assay) was described by Kropinski et al. [33]. Here, we use the acronym of SD/MP (Single Dilutions on Multiple Plates) DAL, as it applies different single dilutions of the specific PL on several different test plates. The phage particles proliferate in the soft agar, while bacteria are fed from the underlying solid agar. The DAL method is generally considered to be the best confirmatory test, as it allows for a precise plaque enumeration and full characterization of individual plaque morphology:

- Plaque diameter;
- Level of transparency/turbidity of the plaques;
- Halo formation and size;
- Motility.

**Figure A5:**
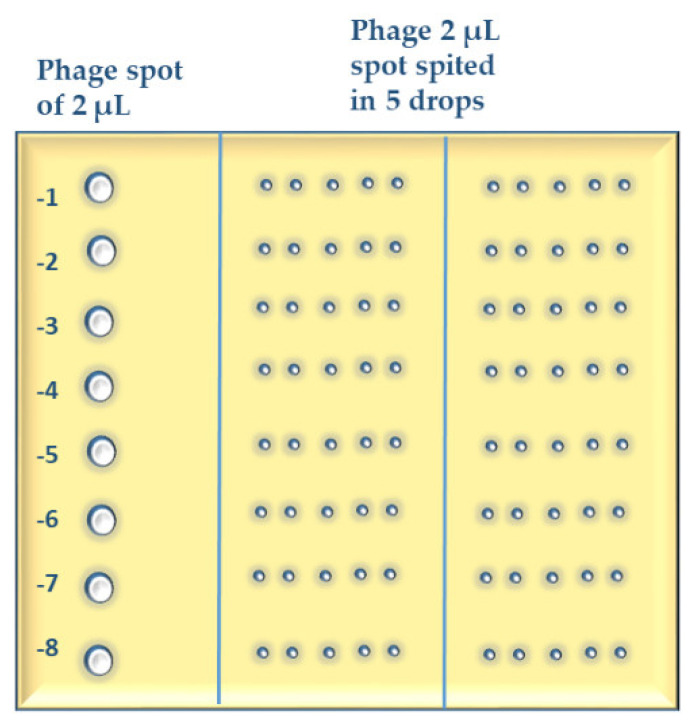
Diagram depicting the splitting of 2 µL spot in 4 smaller drops in the MD/SP method.

Another phage enumeration method described by Kutter et al. (“EOP test”, described below), Kropinski, and Mazzocco et al. (“Drop Plaque Assay”) [33,52,53] is a modification of the SD/MP DAL method and also applies an agar overlay and phage serial dilutions approach, but in this case, multiple dilutions of the phage(s) are displayed on a single plate. For this method, we use the acronym MD/SP (Multiple Dilutions on Single Plates). When dealing with a high number of PLs, microtiter plates can be used to make the dilutions in both the approaches SD/MP and MD/SP DAL (Appendix A, Figure A5).

The disadvantage of the MD/SP DAL method is that it is not precise enough. For more accurate counting and a perfect comparison of the plaque sizes and morphologies on each strain [52], the SD/MP DAL approach is preferred. Another disadvantage is that counting large plaques is difficult, and sometimes it might be better to count the plaques after several hours (4–6 h) [52] instead of 18–24 h, if the tested phage/bacteria growth rate allows for that. If not, an alternative approach consists of using a higher concentration of soft agar (0.8%) and splitting the spot in several smaller drops while applying it on the agar surface (Appendix A, Figure A5).

In addition, some studies have shown that particular phages only reveal clear lysis in the first two dilution spots, with no sign of lytic activity in further dilution spots. The reason for this could be an abortive infection, or “lysis from without” [54], or some other type of bactericidal effect. Some phages do not reveal any lytic activity when spotted [54,55] directly, or in dilutions, but do produce plaques with the SD/MP DAL method. Particularly, the plates with low dilutions of PL often do not display the typically expected results (a clear plate followed by “web pattern-like” lysis zones for the consecutive dilution), while the plates with high dilutions demonstrate clear individual plaques spread through the plate perimeter (personal experience). When only the first two dilutions reveal lytic activity, we recommend further analysis of the PL using SD/MP DAL on the same bacterial strains and/or the repetition of MD/SP on another set of bacterial strain.

#### 2.2.2. Plaque Purification

For plaque differentiation and purification, the most commonly used and described method uses phage streaks [56] on a bacterial lawn, in soft agar, or directly on solid agar as to obtain discrete plaques. We suggest the use of “phage T-streaking” (three-phase streaking) which differs from the “streak assay” used for phage detection. In the T-streaking method, the phage inoculum is streaked over the agar surface in three segments. As such, phage numbers are reduced in each segment, which results in individual phage plaques separated and distanced from each other. In the literature, different numbers of individual plaque passaging rounds are suggested. Usually, three [16] to five [28] passages of individual plaques are considered to be sufficient, but some authors suggest many more passages (e.g., 15–20) [57]. In our opinion (based on practical experience), more than three to five passages should indeed be performed to ensure single plaque proliferation. Moreover, the “phage T-streaking” method could be considered as a preliminary purification method, as it is not accurate enough and used at the very beginning of the plaque purification procedure with five or more repetitions, depending on the given PL. However, to make sure that plaques are the result of a singular phage (clone), several additional steps (five or more) should be performed using the SD/MP DAL method as a confirmatory/validation test. The SD/MP DAL method allows for aggregated plaques to fully spread, creating enough distance between individual plaques on the plates with the different dilutions. An even more advanced approach is to test plaque formation using different host strains in a parallel manner, which allows for the revelation of merged plaques (plaque on plaque).

To validate the plaque purification method as a confirmatory method, the following criteria need to be considered:

- The distance between the plaques (well isolated discrete plaques);
- Different dilutions of phage lysate are applied;
- A certain number of passaging rounds are performed (3–5 final confirmation rounds);
- Several bacterial host bacterial strains are used;
- Several growth media are used.

For practical convenience, mini petri dishes of 35 mm diameter can be used for plaque formation/passaging assays. It is highly recommended to perform a valid plaque purification procedure before moving on to further characterization and activity evaluation.

#### 2.2.3. Bacteria Kits for the Study of Phage Host Range and Efficiency of Plating (EOP)

Bacterial strains for phage host range studies are referred to here as “bacteria kits”. MD/SP DAL is mostly used for the evaluation of EOP [58]. Therefore, MD/SP DAL is often referred to as the “EOP test” [52]. The EOP is the quotient of the phage titer at the terminal dilution on the test strain, divided by the titer of that same phage on its isolation host, expressed in a cardinal number or percentage. As host range studies employ large amounts of bacterial strains, the MD/SP DAL method is usually preferred, as it is repeatable, more automatable, and is less time-, energy- and resource-consuming than SD/MP DAL.

The concept of host range or breadth [15] can be defined in many different ways [18]. It is usually defined as the extent/spectrum of bacterial genera, species, and strains that can be lysed by a phage [52], or which supports phage multiplication [49]. The larger the variety (in terms of genetic and phenotypic profiles) of the bacterial strains that are sensitive to a particular phage or phage mixture, the broader its host range is. The host range is of great importance for the selection of adequate therapeutic phages or phage mixtures [50]. The lytic activity of candidate therapeutic phages should be tested on a large collection of relevant bacteria kits [52]. It is appropriate to aim for the widest possible host range, preferably at the beginning of the selection process [16]. At the same time using a wide range of bacteria kits allows one to identify/reveal more bacterial strains sensitive to the candidate phage and accumulates more EOP data. The bacteria kits should be regularly updated with new isolates originating from relevant clinical environments and geographical areas [52,59,60]. Using bacteria kits that harbor a large genetic variety (composed at least of 100 different genetic profiles) enhances the sensitivity level of the method and makes it more comprehensive as a confirmatory method. Employing widely assorted bacteria kits is important to extend our knowledge and understanding of phage biology and for the potential use of a test phage in different fields (e.g., medicine, food decontamination, or agriculture).

FDA guidance on antibiotic testing requires the testing of at least 100 bacterial strains, and for some species more than 300 strains, with recent clinical isolates accounting for at least 75% of the strains [16]. Following the FDA requirements for bacterial sensitivity testing, bacteria kits of different sizes should be set up locally (laboratory and country level), or on the international level. Biological Resource Centers could function as repositories for host bacteria, harboring the phenotypic and genotypic background necessary for the identification and characterization of phage activity [61]. Important bacterial collections can be found within renowned culture collections such as the American Type Culture Collection (ATCC, https://www.atcc.org/microbe-products#t=productTab&numberOfResults=24 (accessed on 30 March 2022)) or the Deutsche Sammlung von Mikroorganismen und Zellkulturen (DSMZ, https://www.dsmz.de/collection/catalogue/microorganisms/catalogue (accessed on 30 March 2022) [33] and include multidrug-resistant (MDR) strains as defined by the Centers for Disease Control and Prevention (CDC, Atlanta, GA, USA).

At the same time, according to the FDA and some other regulatory bodies for diagnostics (CDC, Forensics), preliminary and confirmatory tests are the main components of systematic qualitative analysis, and this kind of approach needs to be tailored to phage identification, enumeration, and activity evaluation.

### 2.3. Phage Liquid Culturing Method and the Translation of Results

The phage liquid culturing (PLC) method is considered an alternative approach for phage host range and lytic activity measurement [17]. In addition, the lytic activity of phages that are incapable of forming plaques in soft agar could be revealed using this technique [50].

The PLC method, or “Appelmans’ method”, was developed in the 1920s by the Belgian surgeon René Appelmans [46,62]. Initially, the method was developed for phage titration. It uses 10-fold serial dilutions of phage in broth and, after the incubation of each dilution with the host bacteria, the phage titer is evaluated by visual observation. The dilution factor of the last “clear” tube is considered as the phage titer. The modern version of this method uses microtiter plates of different size ranges and multichannel pipettes and is automatable and reproducible, generating digital optical density or colorimetric growth curves, which allows for the testing and comparison of multiple phage/bacteria combinations simultaneously. The simultaneous passaging of different combinations of phage and bacteria is the basis of the phage Host Range Extension (HRE) method that is described in the next section.

The Appelmans’ technique can be used for different purposes:

- Phage enumeration with phage titer expressed as a dilution factor;
- Estimation of the multiplicity of infection (MOI) [10], i.e., the ratio of phages to bacteria, for instance, to set the initial phage/bacterium inoculates for in vitro/vivo studies;
- Evaluation of host range and lytic activity [17];
- Expansion of host range after multiple passaging.

Nowadays, the PLC, or Appelmans’ method, is mostly used and described for the study of phage host range and lytic activity in view of translation to the in vivo context. Different interfering/misleading factors may arise when using this method, such as the growth of phage-mutants [63], the “re-growth” of phage sensitive bacteria [64], and the emergence of temporal immunity to phage lysis [65]. Correspondingly, a rational approach [43] needs to be developed when applying this technique.

Phage-exposed bacterial growth curves have been extensively studied [13,14,27,47,66,67,68,69,70,71,72]. In some cases, the results were translated to in vitro/vivo studies to evaluate the correlation between these studies (Table 1). It is important to mention that the comparison of different in vitro methods (“spot test” or “direct spot test” and PLC) is difficult, as the first method concerns a mostly qualitative assay, even though it could be semi-quantitative under certain conditions (e.g., at low phage titer, when separate plaques are observed within one spot), while the second is a semi-quantitative method. In addition, the bacterial growth conditions (solid versus liquid media) are also different.

#### Host Range Expansion (HRE)

Today, the experimental evolution of individual phages or phage mixtures through serial interactions with one or a mixture of host bacteria is the most used approach to extend the phage host range. Several studies performed in the period 1963–1991 describe the benefit of serial passage experiments (SPEs) that allow for molecular and phenotypic evolution in real time [80]. The changes in phage activity that occurred seemed to depend on the genotypes present in the cocktail at the start of the SPEs [81]. Poullain et al. [82] demonstrated an expansion in the infectivity and growth rate of evolved (the bacterial host is not allowed to evolve) or coevolved (the bacterial host coevolves with its parasite) phages. In phage evolutionary experiments, phages are (serially) transferred from one host culture to a new, phage-naive host culture under defined conditions, and their evolved characteristics are compared with those of their ancestors. Phage evolution on non-evolved hosts is usually accompanied by increasing phage propagation rates. In contrast, in coevolutionary experiments, the phage and its host are transferred together to a fresh culture medium. In this setting, the host is able to continuously coevolve to keep track of phage adaptations, which results in the emergence of different adaptive strategies by the phage. This evolution of phages with their hosts can increase their infectivity ranges [83]. Betts et al. (2013) revealed that bacterial resistance to trained phages emerged at a lower frequency [48]. In 2016, Friman et al. showed that pre-adapting (evolving) phages to *P. aeruginosa* cystic fibrosis bacterial isolates lead to increased pathogen clearance and a lowered resistance evolution as well [83].

Eastern European researchers, particularly in the Republic of Georgia, used the noted Appelmans’ dilutions method [60] for passaging phage mixtures from strain to strain, including both sensitive and resistant bacterial strains, leading to the generation of new variants of phage clones/cocktails lysing a larger range of bacterial cells. This technique was recently applied to pre-adapt a phage for treatment of fracture-related infection due to pandrug-resistant *K. pneumoniae* [84].

Burrowes et al. designed a 96-well plate formatted for Appelmans’ protocol to analyze the individual phages after every 10 rounds of evolution. They showed that starting with a phage cocktail resulted in a larger host-range expansion than when using individual phages, and based on genomic analysis, they observed a recombinatorial origin for output phages with a broadened host-range [60].

The crucial factors for ensuring a rapid host range extension of phages are (1) the use of a phage mixture from the start, which allows recombination to generate sufficient diversity, and (2) the use of both the original bacterial hosts that had been used for phage propagation and an updated collection of clinical bacterial isolates that are resistant to the given phages, as it is important to produce therapeutically useful phages [60].

While Burrowes et al. state that the Appelmans protocol works predominantly via recombination between phages [60,85], Mapes et al. presumed a collateral host-range expansion when they conducted a similar SPE, which they named the “host-range expansion” (HRE) method. However, none of the parental or hostrange extended phages were sequenced, and thus, it was hard to ascertain the exact mechanisms of the occurred changes [86]. In any case, the end products of HRE experiments need to be confirmed and validated by whole-genome sequencing, and tested and proved to be stable, considering the rounds of passaging.

**Figure A6:**
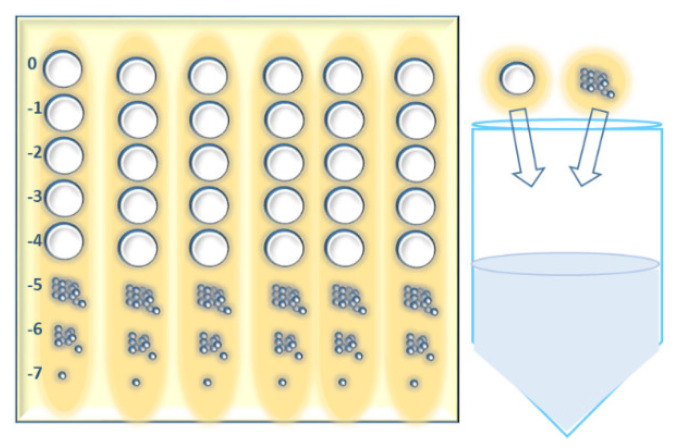
Diagram depicting the “Host Range Expansion (HRE) on agar” method.

Serial passaging for HRE can be performed on agar as well, whenever liquid media are not adequate for demonstration of phage lytic activity. The agar method is more time consuming than the liquid method, but it has the advantage that the obtained phage mixture no longer needs to be processed further for plaque formation (Appendix A, Figure A6).

## 3. Discussion

Many studies refer to existing gaps in standardization and validation of assays/methods documenting phage activity and in the translation of their results to in vivo applications [17,32,34,87]. The definitions of phage host range and test outcomes vary between methodologies [17]. The phage-related experimental measurements described in the phage literature mostly rely on the same principles [13], but without a standardization of tests, it is difficult to correlate in vitro with in vivo results and to interpret disparate findings between studies and laboratories.

**Figure 1:**
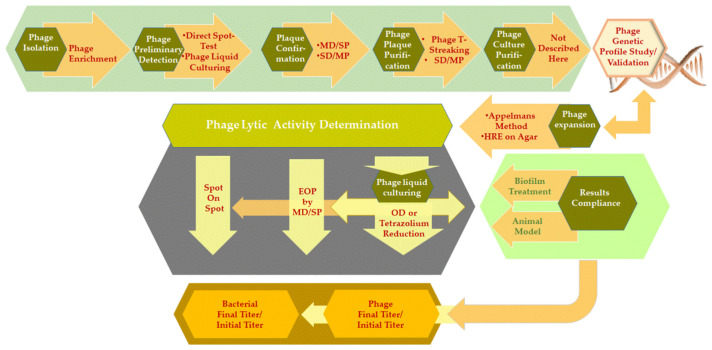
Scheme depicting the chain of methods from phage isolation to study of compliance for phage treatment with in vitro activity evaluations.

Methods determining phage lytic activity (Figure 1) are based on bacterial clearing on either agar or in a liquid medium. In both cases, results can be qualitative—if only a visual observation/evaluation is performed at the end point of the test—or quantitative—if a calculation is made at a particular point(s) in time. However, different quantification methods and principles are used: (i) the determination of the number of phages in pfu/mL, (ii) the determination of bacterial concentration in cfu/mL or optical density (OD), or (iii) the determination of bacterial metabolic activity (e.g., tetrazolium reduction). The results from these approaches can further be used for the calculation of phage yield (ratio final to initial phage titer) or the reduction of bacterial growth (ratio initial to final bacterial concentration). Liquid culturing techniques make it possible to calculate bacterial growth reduction dynamics, but the confirmation of phage growth itself, bacterial re-growth of initially phage-sensitive bacteria, or the selection of phage-resistant bacterial mutants still requires phage plaque and bacterial colony formation on agar.

As we mentioned earlier, the “direct spot test” and “spot test” methods should only be considered as preliminary (qualitative) phage detection (sensitivity) tests, as they are not demonstrating plaque formation, while the MD/SP DAL method can be considered as a confirmation test for phage detection as it demonstrates plaque formation. Since the SD/MP DAL method allows for the most precise phage enumeration and plaque morphology characterization, it could be considered as a confirmatory method for phage enumeration and plaque morphology characterization. Note that the MD/SP DAL method is less time-, material-, and labor-consuming as it allows for the analysis of several phages or phage dilutions on one plate. It would be relevant for host range determination and EOP evaluation. We will not provide a detailed discussion of phage culture purification here, as it is beyond the scope of the present review and would deserve a dedicated paper. However, to ensure that a particular phage lysate (newly isolated or evolved) is a single phage particle product and authentic, it first must go through plaque and then culture purification steps. For adequate phage purification, five or more passages should be performed using the “phage T-streaking” method as a preliminary approach, followed by five or more passaging steps using the SD/MP DAL method, as this method allows for full morphological selection and characterization of phage plaques. Once a particular phage is purified (plaque and culture) using an established and validated procedure, the candidate phage can be submitted to further characterization.

The PLC method has also been put forward as an alternative approach for phage host range measurement [17]. It is frequently used today for the in vitro evaluation of phage-bacterium population dynamics [88] and established as a rapid tool to extend the phage host range [60] or to increase phage lytic activity, as an alternative to the genetic engineering of super phages [89,90].

To analyze phage/bacterium population interaction dynamics in a comprehensive manner, it is advised not to use OD measurement, but to measure the conversion of water-soluble tetrazolium salts, which yields a higher sensitivity and dynamic range. For this, the OmniLog^TM^ system provides a high-throughput capability (4800 phage assays) [73] for the real-time monitoring of bacterial growth dynamics.

The main reason for attempting to standardize phage lytic activity measurements and make them as effective as possible is to be able to correlate phage in vitro traits [22] with therapeutic outcomes. Often, the results of different qualitative or quantitative methods (on agar or in liquid media) are arguably considered to be comparable. The spot qualitative assessment of different phage-bacterium combinations is often scored [17] using cardinal numbers (streak-based method scores of “0”to “+5”), while phage activity determined in liquid media is usually expressed using lysis scores (ranging from 1 to 3) based on OD changes in time. Storms et al. (2020) and Konopacki et al. (2020) developed the phage “virulence index” and “PhageScore” formulas, respectively, which can be used to analyze and compare phage activity and to select phages in a more standardized way. Both formulas are based on bacterial growth curves determined in liquid media [13,78]. However, both formulas need to be tested on a large variety of phage/bacteria combinations in different conditions (described below) to validate the results and to confirm that they are transducible to in vivo applications.

The phage liquid culturing (PLC) method is put forward as the best assay to evaluate phage lytic activity [13,17,78], in comparison to EOP determination using the inherently imprecise MD/SP DAL method. Both methods are performed using different conditions (e.g., medium composition) and are based on different principles with regard to evaluation mechanisms and kinetic recordings. The disadvantage of both methods is that phage titers (pfu/mL) estimated on a “standard” bacterial strain are considered for the evaluation of the effectiveness of the same phage on bacteria kits.

Some relevant phage infection parameters, such as adsorption rate, latent period, and burst size, can be deduced from monitoring phage growth in liquid media [46]. Phage infection parameters depend on bacterial host physiology and nutritional conditions [7,14], which determine bacterial growth itself. Bacterial cells do not experience the same growth conditions on agar as compared to liquid culture [91], and thus, phage infection is also bound to differ. The latent period and burst size of phages are related to the bacterial growth rate [37,92,93]. As such, the phage growth rate is the most important criterion with regard to phage “virulence” [22]. Thus, to correlate phage therapy outcomes with lytic activity (propagation rate and mutant selection), comparable conditions should be applied, i.e., realistic nutritional composition and consistency of media (liquid, semi-solid and solid), incubation times and temperatures, and bacterial host strains. Finally, and most importantly, the initial phage/bacteria ratios should be adjusted separately for each method, and considered further in the integrated evaluating formula for phage virulence or activity capacity as a whole. While optimizing the conditions for each phage/bacterium combination to give the highest possible outcome is feasible in vitro, the in vivo translation of the results is more problematic.

Every phage candidate with the potential to be used in the therapy—be it naturally isolated, with or without expanded activity or host range, genetically engineered or not, or used within a ‘one-size fits all’ or broad-spectrum approach [94]—should ideally be pre-tested in a standardized, comprehensive, and statistically significant way to meet the expectation for successful phage therapy.

Therefore, the question remains as to what should be considered and tested to determine a phage’s potential to reduce the bacterial population at different infection loci.

### 3.1. Bacterial Population and Infection Locus Consistency

Certain bacterial determinants are critical for the outcome of phage/bacteria interactions. In vitro and in vivo phage/mixture testing is most often performed using homogeneous bacterial populations grown either on agar, as planktonic cells in liquid culture, or in biofilms. However, the bacterial composition of the infection loci to be treated with phages (e.g., an infected wound) usually consists of an assembly of different strains belonging the same or different bacterial species [15] and exhibiting different growth modes (planktonic and biofilm). As a result, it is appropriate in certain cases to test a mixture of different bacterial strains to evaluate the lytic activity of phages before treatment. Most important are the virulence factors of bacteria that can hamper phage proliferation. Laboratory conditions (e.g., growth media) are very different from the conditions encountered in vivo [91]. Therefore, bacteria grown using standard laboratory protocols behave differently than those grown in the milieu of an infection (e.g., in a wound bed). For example, *S. aureus* rarely expresses its capsular polysaccharides, which are typical for clinical isolates, when they are grown in the laboratory [95]. *P. aeruginosa* possesses an arsenal of virulence factors enabling it to invade host cells and circumvent host defenses [96], which are not revealed in in vitro conditions. Culture media could be developed by taking into account certain conditions (e.g., pH and viscosity) which allow for the exhibition of virulence factor(s), and thus, a more accurate study of phage behavior. Moreover, to mimic real-life scenarios of localized infections [97], body materials (e.g., sputum, surgical suture, and debris) and fluids (blood/serum, cerebrospinal fluid, bile, etc.) spiked with the relevant bacterial strain(s) could be used as a model.

### 3.2. Phage-Bacteria Ratio

The right phage-bacteria ratio or so-called MOI to achieve complete bacterial lysis over a given period of time in a liquid culture should be determined [15,51]. The ideal cell numbers and MOI are different for each phage [96] and several different studies have revealed that the outcome of phage activity mainly depends on the MOI [98].

The optimal phage-bacteria ratio is correlated with phage = bacteria growth rates, and the balanced combination of phage-bacteria is the main determinant for the successful reduction/delay of the emergence of phage-resistant bacterial mutants. This optimal phage/bacteria ratio can be used in phage-virulence assays or in vitro and animal models.

### 3.3. Phage Mixtures

An appropriate phage mixture or cocktail [5] is believed to be much more effective than single phages to treat infections. This phenomenon is referred to as synergy [28], where the different phages together facilitate the infections [99] of the bacterial population. This synergistic efficacy is mostly based on ensuring coverage of a range of bacterial receptors [100] and to individual phage properties [28]. Conversely, the mixing of phages can also result in less lytic capacity [91] than predicted based on the sum of the coverage and activity of each component phage [36]. Phage components of phage cocktails are typically selected based on as wide as possible, and non-overlapping, host ranges [101] and are mostly mixed in the same proportions. However, it is very important to consider that different phages have different growth rates/adsorption times, and if they are combined in optimally differing titers (pfu/mL), the activity of the phages could be balanced in time. This, together with the right phage-bacteria ratio, may give the most effective outcome of treatment.

Finally, we can conclude that, to date, no validation procedure/format has been developed nor approved by the relevant regulatory authorities for the evaluation, categorization/ranking (preliminary or confirmatory), or documentation of the methods used to assess in vitro and in vivo phage activity in a standardized manner. All of the methods commonly shared and used so far are copied, developed, or modified from manuals and scientific papers, mostly dating from d’Hérelle’s time.

The level of phage virulence as a whole (phage therapy capacity)—host detection, host range, phage-bacteria growth rate, phage-bacterial interaction (including the circumvention of bacterial cell defense systems), phage survival/sustainability, adaption to the host, and invading ability—is associated with conditional factors such as patient age and physiology (e.g., impaired or healthy), concentration of bacteria, temperature, and pH at the infection site. Thus, as phage-bacterial interactions are continuously evolving, so is phage virulence. Phage virulence capacity could be enhanced in vitro by implementing a good understanding of phage-bacterial interactions under certain specific conditions (resembling those at the infection loci). In vitro evaluation of phage activity, using standardized and integrated criteria, is bound to provide a valuable support for in vivo applications. Every selected method should be rational, reliable and appropriate in a particular situation, feasible, and cost effective, considering timelines, labor, and material consumption.

## Figures and Tables

**Table 1:** PLC experiments and translation of the results.

Years	Authors and Study	Results and Outcome
2006	Raya et al. studied: Phage/bacteria dynamics in PLC for T-even phages in aerobic and anaerobic conditions (eclipse, latent period, and burst size at different MOIs). In vivo sheep trials evaluating phage infection control/eradication [67].	Translation of the results of phage/bacteria dynamics in PHL to in vivo sheep trials. Showed the importance of screening for adequate phages using a PE method prior to in vivo studies.
2008	Niu et al.: Studied phage susceptibility testing of Shiga toxin-producing *E. coli* (STEC) isolates using “microplate phage virulence assay”; Classified STECs as extremely, highly, moderately, or minimally susceptible, based on host range at different MOIs; Correlated the evaluated phage lytic capability to a set of other characteristics (based on STEC phage-typing and genotype studies) [14].	Showed that phages exhibiting high growth rates and broad host ranges could be effective as biocontrol agents.
2011	Vandersteegen et al. described studies on the *Staphylococcus aureus* phage infection parameter in two separate papers: First, they used a PLC method; Later, they performed phage-mediated biofilm (biomass) reduction; They did not provide a detailed comparison of the results from both studies [68].	Demonstrated the impact of different MOIs on lytic activity dynamics; Showed phage-mediated biofilm (biomass) reduction after 24 h of incubation; Concluded that using the same phage/bacteria combinations and conditions resulted in comparable phage effectiveness.
2011	Cooper et al. studied *P. aeruginosa* phages’ efficacy with: “qualitative streak” test; “quantitative assay” using the Bioscreen C microbial growth analyzer. Of note, the parameters as phage/bacteria ratio, media, and incubation temperature were different while using these two methods [34].	Only observed similar results for phages exhibiting substantial activity; Assumed that unequal experiment conditions might have contributed to the observed differences in results.
2013	Henry et al.: Studied phage lysis kinetics of eight *P. aeruginosa* phages; Pre-tested the phages using Efficiency of Plating (EOP); Experimented with an in vivo mice model [73].	Demonstrated successful translation of results of EOP and PLC kinetics to an in vivo mice model; Showed that the phages isolated directly on the targeted bacterial host were the most efficient in vivo, supporting a personalized phage therapy approach for optimal treatment outcomes.
2014	Wong et al. Studied the ”lytic spectrum” (host range and susceptibility data) of phages against *Salmonella Typhimurium* at a wide range of MOIs; Performed an in vivo chicken trial [27].	Observed miscorrelation between the in vitro ”lytic spectrum” and the in vivo trial in chickens’ results; Suggested that the in vivo persistence of phages is important to completely eliminate pathogens;
2017	Green et al. performed: In vitro *E. coli* PLC reduction experiments; In vivo infected mice model [74].	Demonstrated an acceptable correlation between in vitro *E. coli* reduction levels and improved health scores in infected mice.
2013–2019	A number of research groups [16,73,75,76] adapted the OmniLogTM system, which monitors bacterial growth based on the respiration rate of growing cells [73]; Studied phage-mediated lysis and (the suppression of) the emergence of bacterial phage resistance [75].	Developed appropriate therapeutic phage cocktails within a short time period; Succeeded in Adaptive Phage Therapeutics’ “Host Range Quick Testing” [16,77].
2018	Xie et al. measured phage host range and “virulence” for 15 Salmonella phages using: The “spot method”; A PLC based assay [17].	Found more correlation for host range evaluations than for “virulence” estimations.
2018	Forti et al. tested a six-phage cocktail against *P. aeruginosa*, which had been designed based on host range and genomic information: In planktonic liquid cultures; In biofilms; In mice; In Galleria mellonella larvae [31].	Showed correlation with MOI; Demonstrated that the cocktail of the six phages was able to lyse *P. aeruginosa* (both in PLC and in biofilms), better than individual phages; Assumed that the phage cocktail could cure acute respiratory infection in mice and treat bacteremia in *Galleria mellonella* larvae; Showed that administration of the cocktail to the larvae prior to bacterial infection provided prophylaxis.
2020	Storms et al. and Konopacki et al., respectively [13,78]: Developed a phage “virulence index” and a “PhageScore”, respectively, both based on bacterial growth curves; Both quantified and compared the virulence of diverse phages individually and in specific combinations, applying different MOIs, Storms et al. used the trapezoidal rule for their “virulence index” formula, which depends on the number of data points creating sub-areas that need to be calculated separately, while Konopacki et al. utilized a continuous function in the calculation area, instead of a coarse, straight-lines growth description, for their “PhageScore” [13,78].	The “PhageScore” allows for a more accurate prediction of the process than the “virulence index” [78]; Both formulas/approaches could be used to evaluate and compare phage activity in view of the selection of candidate therapeutic phages.
2021	Nale et al.: Examined a potential of 21 myoviruses and siphoviruses in vitro against *Salmonella*; Elaborated in vivo infection biocontrol strategy in poultry and swine; Developed a phage cocktail, based on a preliminary defined host range [79].	The phage cocktail showed: High in vitro efficacy; Potential for prophylaxis in a *G. mellonella* larvae model.

## Data Availability

Not applicable.

## Appendix A

### Appendix A.1. Short Outline of the “Phage Isolation Enrichment” Method

1.1 Culture the bacterial strains in 96- or 384-well microtiter plates overnight at an appropriate temperature, in a suitable culture medium.
1.2 Collect 200 (40) µL of each of bacterial suspension from each well of the 96 (384)-well microtiter plates (19.2 (15.36) mL in total correspondingly) and transfer the liquid to a sterile reservoir using a multichannel pipette.
1.3 Add the following ingredients to a sterile container (flask):
  - 360 (288) mL of sewage water.
  - 40 (32) mL of 10× concentrated culture medium (broth).
  - 19.2 (15.36) mL mixture of the bacterial suspensions in the reservoir.
1.4 Incubate the container at 25–28 °C for 18–24 h.
1.5 Centrifuge the (potential) phage lysate at 6000× *g* for 30 min.
1.6 Filtrate the (potential) phage lysate using a 0.45 µm syringe filter.
1.7 Store the supernatant at 4 °C.

### Appendix A.2. Short Outline of the “Spot on Streak” Method

2.1 Make dilutions of the bacterial suspensions in a 96-well-microtiter plate including the following two dilutions: a low concentration containing 1.0 × 10^4^ cfu/mL and an average one containing 1.0 × 10^7^ cfu/mL.
2.2 Apply a drop (20 µL) of each bacterial suspension in the first column of a grid on a square petri dish containing a suitable agar medium, using a multichannel pipette; then roll down each drop to the end of the grid row by using the same pipette and tips or separate disposable loops. Let the bacterial streak dry up in a Biosafety Cabinet (BSC).
2.3 Distribute the phage lysates in a 96-well-microtiter plate or another segmented reservoir according to their foreseen outline on the test agar plate grids. Spot 10 µL of phage lysates on the bacterial streaks in a vertical direction by multichannel pipet.
2.4 Let the spots dry up in a BSC and then incubate the test plates upside down at a temperature of 25–28 °C (which should be lower than the standard incubation temperature for the considered bacterial strains) for 18 h.

### Appendix A.3. Short Outline of the “Spot on Spot” Method

3.1 Repeat the first step of the “spot-test on streak” method.
3.2 Spot 10 µL of the bacterial suspensions in the first column of the grid. Let the bacterial spot dry up in a BSC.
3.3 Spot 5 µL of phage lysate over the bacterial spot.
3.4 Repeat step 2.4. of the “spot on bacterial streak” method.

### Appendix A.4. Short Outline of the “Streak on Streak” Method

4.1 Apply phage lysate drops (20 µL) in the first column of a grid on a square petri dish containing a suitable agar medium, using a multichannel pipette; then roll down each drop to the end of the grid row by using the same pipette and tips or separate disposable loops. Don’t allow phage streaks to dry up before bacterial suspensions are applied.
4.2 Streak 10 µL of bacterial suspensions over the phage streaks. Let the bacteria/phage streaks dry up in a BSC.
4.3 Repeat step 2.4. of the “spot on streak” method.

### Appendix A.5. Short Outline of the MD/SP (Multiple Dilutions on Single Plates) Method

5.1 Make ten-fold serial dilutions of phage lysate(s) in 96-well microtiter plates (add 20 µL of phage suspension to 180 µL of phosphate buffered saline) typically up to 10^−8^.
5.2 Mix 300 µL of bacterial suspension of an OD that is preliminary adjusted for each host strain or species with up to 8 mL of molten soft agar (0.7% or 0.8% suitable agar 46 °C) in a 15 mL tube and pour the mixture onto pre-prepared square petri dishes with 1.5% agar medium. Use 0.8% soft agar for phages that form large plaques. Let the plates dry up for 10–15 min in a BSC.
5.3 Spot 2 µL of each phage dilution onto the soft agar surface across the column of the plate grid (six columns on a square petri dish) using a multichannel pipette. Make three repetitions of each test phage. In case of phages with large plaques, make a three-column grid on a square petri dish and split the 2-µL-spot in 4 smaller drops while applying on the agar surface.
5.4 Use standard phage dilutions (with known titer), on each test plate (whenever possible) as control for the titration.
5.5 Let the test plates dry in a BSC and incubate them upside down at 28–32 °C (depending on the host bacteria) for 18–24 h.
5.6 After incubation, calculate the average number of plaques for the different dilutions and repetitions and multiply them by 500 to obtain the number of plaques in 1 mL. The phage titer (pfu/mL) is the number of plaques in 1 mL multiplied by the reciprocal of dilution.

### Appendix A.6. Short Outline of “Host Range Expansion (HRE) on Agar” Method

6.1 Make phage mixture dilutions as described in the MD/SP method (step 5.1.).
6.2 Make bacterial streaks lines of 30 µL as described in the “spot on streak” method (steps 2.1.–2.2.). Six lines in total are made on a square petri dish.
6.3 Spot 10 µL of each phage mixture dilution (from zero dilution to 10^−7^) lengthways on the bacterial lines.
6.4 Repeat step 2.4. of the “spot on streak” method.
6.5 After incubation, cut out all agar zones with different clearings (from clear to separate plaques). If there is no sign of phage activity on a particular strain, cut out the agar from the zero dilution zone only.
6.6 Collect all agar cuts in one container and add a volume of phosphate buffered saline corresponding to 3–5 mL per agar cut.
6.7 Stir the container with its content for 1–1.5 h at 400 min^−1^ and then centrifuge at 6000× *g* for 30 min.
6.8 Filtrate the supernatant using a 0.45 µm syringe filter.
6.9 Repeat the passaging rounds until the expected phage host-range extension is obtained.
